# Revolutionizing the treatment for nasopharyngeal cancer: the impact, challenges and strategies of stem cell and genetically engineered cell therapies

**DOI:** 10.3389/fimmu.2024.1484535

**Published:** 2024-10-10

**Authors:** Chin-King Looi, Ee-Mun Loo, Heng-Chee Lim, Yik-Ling Chew, Kok-Yong Chin, Shiau-Chuen Cheah, Bey Hing Goh, Chun-Wai Mai

**Affiliations:** ^1^ School of Postgraduate Studies, International Medical University, Kuala Lumpur, Malaysia; ^2^ Faculty of Pharmaceutical Sciences, UCSI University, Kuala Lumpur, Malaysia; ^3^ Advanced Genomics Laboratory, AGTC Genomics, Kuala Lumpur, Malaysia; ^4^ Department of Pharmacology, Faculty of Medicine, Universiti Kebangsaan Malaysia, Kuala Lumpur, Malaysia; ^5^ Faculty of Medicine and Health Sciences, UCSI University, Port Dickson, Negeri Sembilan, Malaysia; ^6^ Sunway Biofunctional Molecules Discovery Centre, School of Medical and Life Sciences, Sunway University Malaysia, Bandar Sunway, Selangor Darul Ehsan, Malaysia; ^7^ Biofunctional Molecule Exploratory Research Group, School of Pharmacy, Monash University Malaysia, Bandar Sunway, Selangor Darul Ehsan, Malaysia; ^8^ College of Pharmaceutical Sciences, Zhejiang University, Zhejiang, China

**Keywords:** nasopharyngeal carcinoma, stem cell, chimeric antigen receptor (CAR)-T cell, engineered T cell receptor-T (TCR-T) cell, tumour-infiltrating lymphocytes (TIL), natural killer (Nk) cell

## Abstract

Nasopharyngeal carcinoma (NPC) is a distinct malignancy of the nasopharynx and is consistently associated with the Epstein-Barr virus (EBV) infection. Its unique anatomical location and complex aetiology often result in advanced-stage disease at first diagnosis. While radiotherapy (RT) and chemotherapy have been the mainstays of treatment, they often fail to prevent tumour recurrence and metastasis, leading to high rates of treatment failure and mortality. Recent advancement in cell-based therapies, such as chimeric antigen receptor (CAR)-T cell therapy, have shown great promise in hematological malignancies and are now being investigated for NPC. However, challenges such as targeting specific tumour antigens, limited T cell persistence and proliferation, and managing treatment-related toxicities must be addressed. Extensive research is needed to enhance the effectiveness and safety of these therapies, paving the way for their integration into standard clinical practice for better management of NPC and a better quality of life for human health.

## 1 Introduction

Nasopharyngeal carcinoma (NPC) is an undifferentiated form of squamous cell carcinoma (SCC) formed from the uncontrolled growth of cells in the epithelium layer of the nasopharynx. While globally rare, it is prevalent in Southern China, Southeast Asia, North Africa and the Middle East. Among the Cantonese population in Southern China, the incidence is approximately 25-50 cases per 100,000, compared to less than 1 per 100,000 in other parts of the world (1).. This low incidence contributes to limited research and fewer diagnostic advancements compared to other cancer types, making it challenging to effectively identify and diagnose NPC. In 2020, the International Agency for Research on Cancer (IARC) reported 133,354 cases and 80,008 deaths of NPC worldwide, with 62,444 (46.8%) cases in China, and 36,747 (27.6%) cases in the rest of Southeast Asia (2). These findings highlight persistent disparities in NPC incidence and mortality globally, underscoring the urgent need for improved diagnostic tools and treatment strategies to address the NPC burden in affected regions and countries. Notably, NPC incidence may vary based on gender, age, Epstein-Barr virus (EBV) infection, and risk factors such as smoking, alcohol consumption, occupational exposures and dietary consumption of Cantonese-style salted fish (1, 3).

The conventional treatment modalities for NPC include chemotherapy, radiotherapy (RT) (4), and immunotherapeutic strategies (5). While emerging evidence highlights that surgery or nasopharyngectomy is effective in the salvage of recurrent tumours or metastasis after primary RT or when other treatments have failed (6), it has a significant disadvantages in treatment of primary NPC. The anatomical location of the nasopharynx limits the visual field, making surgical access difficult. Moreover, surgery is risky due to the proximity to critical structures such as the internal carotid artery, spinal cord, and cranial nerves, which can cause serious consequences if accidentally injured. Additionally, patient’s quality of life (QOL) has been shown to deteriorate significantly with surgical treatment. Therefore, these limitations have made non-surgical treatments the primary options for NPC (7). While RT has been the primary treatment since 1965, with a high 5-year overall survival rate of up to 90% with RT alone for Stage I NPC patients, treatment with RT has been associated with severe acute and late toxicities (4). Delivering a high radiation dose to targets while protecting radiosensitive organs such as the brain stem, spinal cord, temporal lobes, middle and inner ears, and parotid glands, which are anatomically surrounded by the nasopharynx, is particularly challenging (8). Additionally, chemotherapy can increase susceptibility to infection due to immunosuppression and may cause systemic adverse effects like vertigo and hair loss. Chemotherapy resistance is also a significant issue that often leads to therapeutic failure (9). Despite recent advancements in immunotherapies, especially immune checkpoint blockade therapy, treatment responses vary significantly among patients, and high rates of relapse with poor prognosis and treatment unresponsiveness remain significant challenges in improving patient’s outcomes (10).

In recent years, cell-based therapies, particularly those involving genetically modified T cells with chimeric antigen receptors (CAR), have shown promise in treating hematologic malignancies (11) and are being evaluated for NPC (12, 13). NPC is an epithelial malignancy closely associated with EBV infection and is characterised by intense infiltration of immune cells. Despite the abundance of tumour-infiltrating lymphocytes (TILs), many of these cells are highly activated and exhausted, expressing exhaustion markers such as lymphocyte activation gene (LAG-3), cytotoxic T-lymphocyte-associated protein 4 (CTLA-4), and programmed cell death 1(PD-1) (14), while high programmed death ligand 1 (PD-L1) expression on NPC cells promotes immune evasion (15). In addition, EBV expresses a series of latent viral genes, including latent membrane proteins (LMP1 and LMP2), Epstein–Barr nuclear antigen (EBNA1), BamH1 A fragment rightward reading frame 1 (BARF1), non-polyadenylated and non-protein coding small EBV RNAs (EBER1 and EBER2), BamHI A rightward transcripts (BARTs) and BART microRNAs (miR-BARTs) (16). These genes support the growth of infected cells and serve as attractive targets for immunotherapy and cell-based therapies, such as CAR-T. LMPs are highly immunogenic and activate nuclear factor kappa B (NF-κB) and signal transducer and activator of transcription 3 (STAT3) signalling pathways, leading to the expression of various downstream targets involved in chronic inflammatory responses. These include interleukins (IL-6, IL-10, TNF-α), chemokines (CCL4, CCL5, CXCL10), vascular endothelial growth factor (VEGF), and hypoxia-inducible factor (HIF)-1α, all of which are involved in immune evasion, cell growth and proliferation, invasion, metastasis, and epithelial-mesenchymal transition (EMT) (17). Targeting EBV-specific antigens with cell-based therapies may offer long-lasting effects and reduce the risk of recurrence, with fewer side effects compared to conventional treatments (18). Inspired by the success of adoptive EBV-targeted cytotoxic T lymphocyte (EBV-CTL) therapy in other EBV-associated diseases (19, 20), anti-EBV cell-based strategies are gaining attention as potential treatments to improve NPC prognosis (21–23).

In this review, we delve into the potential of cell-based therapies as the next frontier in the treatment of NPC. We critically discuss the general mechanism of action and breakthroughs in cellular therapies and provide examples of utilising engineered immune cells in targeting NPC. Additionally, we summarise findings from both completed and ongoing clinical trials related to adoptive cell therapies against NPC and other cancers. We also address the limitations associated with cellular therapies and suggest integrating these therapies with conventional treatments to improve safety and effectiveness, aiming to enhanced outcomes for advanced NPC patients.

Despite the promise of developing new agents that target essential cellular pathways in cancer progression, most advanced cancer patients have experienced relatively short-term benefits. Among the emerging biologic therapeutics, cell-based therapy, also known as cell therapy or cellular therapy, holds potential to treat many intractable human diseases, including cancer (24). The first practice of cell therapy was introduced in the late 1950s by E. Donnall Thomas, who pioneered the use of hematopoietic stem cell (HSC) transplantation for the treatment of leukemia (25). Since then, the field of cell therapy has continuously progressed, with ongoing investigations focused on clinical safety and efficacy. Notably, the Food and Drug Administration (FDA) has approved Provenge (sipuleucel-T; Dendreon), an autologous cellular immunotherapy for metastatic castration-resistant prostate cancer in 2010 (26). In 2017, the FDA approved two genetically modified autologous T cell immunotherapies, Kymriah (tisagenlecleucel) and Yescarta (axicabtagene ciloleucel), for acute lymphoblastic leukaemia (ALL) (27), and relapsed or refractory large B cell lymphoma (LBCL) (28), respectively. Additionally, BRG01, an engineered, allogeneic, EBV-targeting T cell therapy, has recently received both fast-track and orphan drug designation from the FDA for EBV-positive relapsed/metastatic NPC (29). As highlighted in the Cell Therapy Market Size, Share, & Trends Analysis Report, the global cell therapy market size valued at USD 4.77 billion in 2022, is expected to grow at a compound annual growth rate of 16.5% from 2023 to 2030. This underscores the increasing adoption and demand for cell therapy solutions worldwide, opening a new era of therapy for human diseases.

Cell therapy can be generally classified into two categories: (i) stem cell-based and (ii) non-stem cell-based. It typically involves the use of autologous or allogenic cells and may incorporate genetic engineering or manipulations in formulation to achieve therapeutic effect (24).

## 2 Stem cell-based therapies

When comes to cell therapy, it is logical to explore the therapeutic potential of stem cells. Stem cells can be found in both embryos and adult cells. They are undifferentiated cells capable of self-renewal and differentiation into specialised cell types based on their developmental potency. This potency gradually reduced from totipotency (in the zygote and early embryonic cells) to pluripotency (in embryonic stem cells, ESCs), multipotency (in hematopoietic stem cells, HSCs), and unipotency (in the dermatocytes) (30). In cancer treatment, stem cells used include adult stem cells (ASC), and pluripotent stem cells (PSC), Different stem cells exhibit varying capabilities in proliferation, migration, and differentiation, defining their suitability for antitumour therapy (31).

### 2.1 Adult stem cells

Adult stem cells (ASCs), also known as somatic or resident stem cells, are undifferentiated cells found in various tissues with limited self-renewal ability and differential potential (32). Examples of ASCs include HSCs, neural stem cells (NSCs), and mesenchymal stem cells (MSCs).

HSCs are predominantly found in bone marrow, peripheral blood (PB) and umbilical cord blood (UCB). They play a crucial role in regenerating blood cells and treating haematologic malignancies through HSC transplantation (HSCT), also known as bone marrow transplantation (33). This procedure, pioneered by E. Donnall Thomas in the late 1950s, involves infusing autologous or allogeneic stem cells to reconstruct a functional hematopoietic system (25). HSCT is now a standard treatment for various hematologic malignancies and have also been explored in solid tumours as immune cell progenitors, exhibiting synergistic effects with adoptive T cell immunotherapy and dendritic cell vaccines. Studies show that HSCs facilitate T cell trafficking, augment immunological rejection of invasive tumour cells, and can differentiate into immune-stimulating dendritic cells (DCs) within the TME (34). These findings highlight the multifaced role of HSCs not only as stem or progenitor cells for cell regeneration, but also as cells capable of synergistically enhancing the efficacy of immunotherapy against solid cancers. However, till today, there is limited evidence of HSCs in treating NPC patients. And thus, HSCs may have theoretical role against NPC, but no clinical study has confirmed its efficacy.

NSCs are self-renewable stem cells in the central nervous system, capable of differentiating into astrocytes, oligodendrocytes, and neural cells (35). Engineered NSCs have shown promise in treating brain cancers due to their ability to migrate to malignant brain masses (36). While the mechanism of NSC homing to tumour is not fully understood, hypoxia has been identified as a key factor. HIF-1α in the TME activates NSC chemoattractant production, such as cytokines, chemokines, and growth factors (37). This migratory behaviour positions NSCs as an effective tumour eradication therapy (38, 39). Similar to HSCs, currently no clinical study has confirmed NSC can eradicate NPC, and thus further study is warranted to investigate whether the NSC has its clinical value for NPC patients.

MSCs, derived from sources such as bone marrow, adipose tissues, PB, placenta, and umbilical cord, playing roles in tissue repair and regeneration due to its primary, non-specialised, non-haematopoietic, with rapid proliferation and differentiation potential. MSCs also exhibit immunomodulatory and anti-inflammatory properties, and are non-immunogenic, making them suitable for transplantation without immunosuppression. For example, bone marrow-derived MSCs inhibited CTL activation by downregulating natural killer group 2 member D protein (NKG2D) receptor expression and increasing the production of immunosuppressive factors (40). MSCs also promote Tregs differentiation to suppress allogeneic T cell response (41). MSCs also suppress tumour growth by modulating cell cycle signalling pathways and inducing apoptosis (42, 43). However, MSCs is a double edge sword in cancer therapy, in which MSCs can differentiate into cancer-associated fibroblasts (CAFs) in response to TGF-β released from tumour cells, leading to the promotion of tumour growth and progression (44, 45).

It is also worth noting that MSCs release exosomes that facilitate cell communication (46) and modulate disease progression (47, 48). In NPC, MSC-derived exosomes are associated with elevated expression of fibroblast growth factor (FGF)-19, enhancing tumour growth, migration, and metastasis through the activation of the FGF19-fibroblast growth factor receptor 4 (FGFR4)-dependent ERK signaling cascade and promotion of EMT (49). However, some reports suggest that these exosomes can inhibit tumour growth by reversing its therapy resistance. For instance, MSC-derived exosomes with overexpressed tumour suppressor microRNA-34c-5p (miR-34c) have shown potential therapeutic value in reversing radioresistance and inhibiting malignant behavior in NPC cells (50). Similarly, exosomes from engineered human MSCs with elevated expression of microRNA-125a-3p (miR-125a) demonstrated the ability to suppress migration and vasculogenic mimicry formation in NPC cells, suggesting potential applications in anti-angiogenic therapy for cancer treatment (51).

In summary, HSC and NSC are currently still under investigation whether its potential can be translated to bedside for NPC patients. The dual role of MSCs as tumours promoter or suppressor (**Table 1**), may depend on tumour types, treatment doses, and duration. The role of MSCs as antitumour agents or therapeutic targets for NPC remains debated, and current evidence is insufficient to support the clinical application of MSC in NPC treatment. Further studies are needed to understand the complex interactions between MSCs and cancer, particularly the role of MSC-derived exosomes in carcinogenesis and therapy resistance.

**Table 1:** Dual roles of MSC-derived exosomes in human cancers.

	Cancer types	Source of exosome	Exosomal cargo/pathway	Findings	Reference
**Tumour promoter**	Gastric cancer	UC-MSCs	Activated Akt pathway	Increases expression of mesenchymal markers and promotes angiogenesis via Akt pathway.	(52)
	AML	BM-MSCs	S100A4	Promotes cell proliferation, invasion, and chemoresistance.	(47)
	Breast cancer	Human and mouse tumour-educated MSCs	TGF-β, C1q, & semaphorins	Accelerates tumour progression by inducing differentiation of M-MDSCs into M2-polarized macrophages.	(48)
	NPC	BM-MSCs	FGF19	Promotes tumour progression via FGF19-FGFR4-dependent ERK signaling cascade and EMT.	(49)
**Tumour suppressor**	NPC	MSCs	miR-34c	Suppresses malignant behaviour and enhances radiosensitivity of NPC cells.	(50)
	NPC	MSCs	miR-125a	Attenuates migration and vasculogenic mimicry formation in NPC cells.	(51)
	Gastric cancer	BM-MSCs	miR-1228	Inhibits angiogenesis and tumour progression by downregulating MMP14 expression.	(53)
	Pancreatic cancer	BM-CSCs	Galectin-9 siRNA, oxaliplatin	Suppresses macrophage polarization and Tregs suppressive activity, while increasing CTL recruitment.	(54)

UC-MSCs, Umbilical cord-derived mesenchymal stem cells; Akt, Protein kinase B; AML, Acute myeloid leukemia; BM-MSCs, Bone marrow-derived mesenchymal stem cells; S100A4, S100 calcium binding protein A4; TGF-β, Transforming growth factor beta; C1q, Complement component 1q; M-MDSCs, Monocytic myeloid-derived suppressor cells; NPC, Nasopharyngeal carcinoma; FGF19, Fibroblast growth factor 19; FGFR4, Fibroblast growth factor receptor 4; ERK, Extracellular signal-regulated kinase; EMT, Epithelial-mesenchymal transition; miR-34c, microRNA-34c-5p; miR-125a, microRNA-125a-3p; miR-1228, microRNA-1228; MMP14, Matrix metalloproteinase 14; siRNA, Small interfering RNA; CTL, Cytotoxic T lymphocytes.

### 2.2 Pluripotent stem cells

Pluripotent stem cells (PSCs) can proliferate infinitely and differentiate into various specialised cell types. ESCs and induced pluripotent stem cells (iPSCs) are two types of human PSCs with significant implications for regenerative medicine and clinical research (55). The first human ESC line was derived by James Thomson and his team from *in vitro*-fertilised human embryos in 1998 (56). Due to ethical issues with ESCs (57), Takahashi and Yamanaka generated iPSCs from mouse embryonic and adult human somatic cells in 2006 and 2007, respectively (58, 59). These cells exhibit similar properties to ECSs, including pluripotent status, morphology, growth properties, surface antigen, gene expression, and telomerase activity, highlighting their potential in medical applications.

iPSCs can be genetically engineered into T cells or NK cells, which can effectively target and eradicate tumour cells. The generation of iPSC-derived T cells involves the induction of mesoderm specification and hematopoietic commitment, followed by T cell differentiation. Briefly, iPSCs collected from healthy donors are co-cultured on murine cell lines (C3H/10T1/2 or OP9) with morphogens, such as bone morphogenetic protein 4 (BMP4), VEGF, and fibroblast growth factor (FGF) to induce mesoderm specification. These cells are then co-cultured in a cocktail of cytokines to generate CD34^+^ HSCs (60). The CD34^+^ hematopoietic progenitors are transferred onto OP9 overexpressing the Notch ligand Delta-like 1 (DLL1) or DLL4 (OP9-DLL1 and OP9-DLL4) feeder cells to induce T cell development in the presence of cytokines (60). Similarly, iPSC-derived NK cells are generated from CD34^+^ hematopoietic progenitor cells using feeder-dependent culture method (61). However, murine-derived stroma feeder cells pose risks of cross-species contamination and complicate quality control due to the reliance on different serum and basal media. Therefore, feeder-free methods, such as using immobilized-DLL4 protein or DLL4-µbeads with lymphopoietic cytokines, have successfully induced T cell differentiation without feeder cells (62, 63). Researchers have also generated rejuvenated iPSC-derived antigen-specific T cells that retain the same antigen specificity and cytotoxicity as the original T cells, with higher proliferative capacity and reduced exhaustion markers (64, 65). iPSC-derived human papilloma virus (HPV)-specific CTLs have demonstrated more potent and sustained cytotoxic activity against cancer cells compared to original peripheral blood CTLs (66). Likewise, iPSC-derived EBV-specific CTLs persisted as central memory T cells *in vivo* for at least 6 months, continuously targeting EBV-associated lymphoma cells (67). Another study demonstrated that incorporating an inducible caspase-9 (iC9)-based suicide system into iPSC-derived EBV specific CTLs effectively suppressed tumour growth *in vivo* without compromising antigen-specific killing activity. This system also reduced cytokine release syndrome (CRS), enhancing the safety of T cell therapy (68).

NK cells derived from iPSCs exhibit greater cytotoxicity against a wide variety of cancers. By combining embryoid body (EB) formation with membrane-bound IL-21-expressing artificial APCs, functional NK cells can be efficiently produced from iPSCs. Briefly, iPSCs collected from healthy donors are grown under feeder-free conditions for a week, spun to aggregate into EBs, and cultured in feeder-free media with morphogens to induce the formation of HSC progenitors. The hematopoietic progenitor-containing EBs are then transferred to a feeder-free plate with NK differentiation media containing cytokines (namely IL-3, IL-15, IL-7, SCF, and Flt3L) to generate iPSC-derived NK cells (69). This method offers a safe and robust platform for producing clinically applicable immune cells. Preclinically, iPSC-derived NK cells have demonstrated effective cytotoxic responses against a variety of tumours in xenograft models. For example, iPSC-derived NK cells combined with anti-PD-1 therapy showed synergistic effects in ovarian cancer, delaying tumour progression and enhancing T cell recruitment and inflammatory cytokine production, transforming a “cold” TME into a “hot” TME (70). Although iPSC-NK cell therapy has shown promising results, it has yet to receive FDA approval. A recently completed phase I clinical trial (NCT03841110) involved evaluating the safety and efficacy of FT500, an off-the-shelf iPSC-NK cell product, in combination with one of three immune checkpoint inhibitors (nivolumab, pembrolizumab, or atezolizumab) in patients with solid tumours, including HNSCC (71). The administration of six doses of FT500 cells was well tolerated, with no serious adverse events, such as graft-versus-host disease (GvHD), cytokine release syndrome (CRS), neurotoxicity (NT), or host immune rejection. Nine out of 13 patients achieved stable disease, and one patient with Hodgkin lymphoma refractory to prior anti-PD-1 therapy experienced a 58% reduction in tumour size following the combination therapy (71). This trial provides clinical support for the high tolerability and potential antitumour efficacy of allogeneic iPSC-NK cell therapy.

Based on the evidence above, we advocate that iPSCs as a platform to provide an unlimited source of rejuvenated T cells or NK cells-based therapy. Such cell-based therapy holds promise as a safe and effective method for targeting NPC and other cancers. Further studies are warranted to investigate the possibility of introducing the CARs or transgenic T cell receptors (TCRs) into the iPSC-derived immune cells to further enhance their tumour-targeting capabilities while suicide gene-based safeguard system to ensure its safety.

## 3 Non-stem cell-based therapies

Non-stem cell-based therapies typically involve the use of somatic cells isolated from the human body and undergo processes such as propagation, expansion, and selection. These cells are subsequently administered to patients for various purposes, including curative, preventive, or diagnostic applications (72). Examples of non-stem cell-based therapies include chimeric antigen receptor (CAR)-T cells, engineered T cell receptor-T (TCR-T) cell therapy, tumour-infiltrating lymphocytes (TILs) therapy, NK cell therapy, CAR-NK cell therapy, aimed at enhancing their ability to recognise and eliminate malignant cells (72). **Tables 2**, **3** provide a summary of preclinical and clinical studies on cellular therapies for NPC.

**Table 2:** Summary of preclinical studies on cellular therapies for NPC.

Intervention	Target	Aims of the study	Outcomes	Reference(s)
CAR-T	LMP1	To assess the efficacy of second-generation CAR on targeting the LMP-1 protein to improve EBV-targeted T cell therapy	HELA/CAR-T cells exhibited specific recognition of LMP1-positive NPC cells. They induced efficient killing via the production of IL-2 and IFN-γ in a LMP1 specific manner.	(73)
		To enhance the antitumour efficacy of HELA/CAR-T cells using a third-generation CARs	HELA/137CAR produced larger quantities of IFN-γ and IL-2 and demonstrated superior antitumor activity and long-term persistence in an LMP1-positive NPC xenograft model compared to HELA/CAR.	(74)
TCR-T	LMP1	To develop and evaluate a novel TCR gene transfer regimen to rapidly and reliably generate T cells specific to EBV-encoded LMP1	T-cells engineered with LMP1-specific TCR can recognise and elicit specific cytotoxicity towards LMP1-expressing tumour cells with increased production of IL-2 and IFN-γ.	(23)
	LMP2	To evaluate the effectiveness of different TCR promoters in lentiviral vectors for the transduction of LMP2-specific TCRs into activated T cells, with the goal of developing a universal, MHC-restricted approach for treating EBV-associated tumours	Lentiviral vectors containing the Vβ 6.7 promoter were found to be optimal for TCR gene expression, maintaining expression for up to 7 weeks. These transduced T cells effectively recognized EBV antigens, demonstrated by their cytotoxicity and IFN-γ secretion. Additionally, mice infused with these cells showed significant resistance to LMP2-positive NPC cells.	(75)
		To develop a TCR gene transfer method to quickly generate T cells specific for LMP2 and evaluate their effectiveness in inhibiting LMP2-positive tumour growth	The optimized HLA A*1101-restricted TCR led to the generation of high-avidity T cells with strong antigen-specific functions, such as proliferation, cytotoxicity, and cytokine release. These engineered T cells effectively inhibited LMP2-positive tumour growth in a mouse model and lysed LMP2-expressing NPC cells from advanced NPC patients.	(76)
NK cells	LMP2	To investigate the cytotoxic function of NK cells in EBV-associated epithelial malignancies	LMP2A-mediated upregulation of F3 through PI3K/AKT signaling pathway inhibits the antitumour function of NK cells. Inhibition of F3 restored NK cell cytotoxicity and showed therapeutic efficacy when administered with adoptive NK cells.	(77)
	–	To investigate the effect of radiotherapy on the killing of NPC cells by NK cells in combination with PD-1 inhibition	Radiotherapy sensitized NPC cells to NK cell killing and increased the expression of PD-L1 in NPC cells and the PD-1 in NK cells. Blocking the PD-L1/PD-1 checkpoint further enhanced the cytotoxicity of NK cells.	(78)
	LMP1	To elucidate how EBV infection impairs NK cell cytotoxic function in NPC and to explore the therapeutic potential of combining B7-H3 deletion on tumour cells with anti-PD-L1 treatment to restore NK cell-mediated antitumour activity	LMP1-upregulated B7-H3 expression suppresses NK cell cytotoxicity via the PI3K/AKT/mTOR signaling pathway. Knockdown of B7-H3 in tumor cells, combined with anti-PD-L1 treatment, restored NK cell function and enhanced cytotoxicity against NPC cells.	(79)

CAR, Chimeric antigen receptor; LMP, Latent membrane protein; EBV, Epstein-Barr virus; IL-2, Interleukin-2; IFN-γ, Interferon-γ; TCR, T cell receptor; MHC, Major histocompatibility complex; HLA, Human leukocyte antigen; NK, Natural killer; F3, Coagulation factor III; PD-1, Programmed cell death receptor 1; PD-L1, Programmed cell death ligand 1; PI3K, Phosphoinositide 3-kinase; Akt, Protein kinase B; mTOR, Mammalian target of rapamycin.

**Table 3:** Summary of clinical studies on cellular therapies for NPC.

Intervention	Target	Clinical trials ID	Phase	Status	Outcome measure	Efficacy & Long-term safety	Limitations	Reference(s)
CAR-T	EpCAM	NCT02915445	I	Active, not recruiting	To determine the response rate and assess treatment-related adverse events/dose limiting toxicity of EpCAM-CAR-T cells	Two patients had PR and three showed >23 months of PFS. No CRS event was reported. 50% (6/12) of the enrolled patients experienced self-remitted grade 1/2 toxicities, one patient (8.3%) experienced reversible grade 3 leukopenia.	Limited number of enrolled patients to confirm the clinical benefit of infused T cells.	(80)
	LMP1	NCT02980315	I/II	Unknown status	To evaluate the safety of the designed LMP1-CAR-T cells and determine whether the CAR-T cells are effective in the treatment of EBV-associated malignant tumours	–	–	–
TCR-T	EBV	NCT05587543	I	Recruiting	To compare the safety and efficacy of EBV TCR-T versus CAR-T cells in the recurrent/refractory EBV-positive NPC	–	–	–
	LMP2	NCT04509726	I/II	Recruiting	To assess the maximum tolerated dose of LMP2-specific IL-12-secreting TCR-T cells in EBV-positive metastatic/refractory NPC patients	–	–	–
		NCT03925896	I	Unknown status	To determine the safety and efficacy of EBV TCR-T cells in the treatment of recurrent/metastatic NPC with positive EBV infection in the Chinese population	–	–	–
	LMP1 LMP2 EBNA1	NCT03648697	II	Unknown status	To investigate the safety and tolerability of EBV-TCR-T cell therapy in subjects with NPC who had received prior therapy for their disease, but their disease has progressed or relapsed	–	–	–
EBV-CTLs			I		To evaluate the safety and efficacy of autologous EBV-specific CTLs in treating stage IV NPC that was refractory to conventional treatments.	Patients who received a median of 10 CTL infusion were well tolerated with the induction of LMP-2-specific immunologic responses. Control of disease progression was obtained in six of 10 patients (two with PR and four with SD).	EBV-CTLs monotherapy may face limitations similar to chemotherapy, where malignant clones with low EBV antigen expression might evade CTL-mediated killing. This suggests the need for combining EBV-CTLs with other conventional therapeutic modalities in future studies.	(81)
			I/II		To assess the toxicity, efficacy, specificity and expansion of infused CTLs for recurrent/refractory NPC patients	EBV-CTLs infusion was well-tolerated and provided significant long-term clinical benefits, especially for patients with locoregional disease, with OS rates of 87% at 1-year and 70% at 2-years. However, it showed limited antitumour activity in metastatic disease and was associated with a higher risk of disease progression.	Lack of a clear impact of EBV-CTL antigen specificity or *in vivo* expansion on treatment outcomes, and the results may not be generalizable due to the small sample size.	(82)
	LMP2 EBNA1	NCT02578641	III	Completed	To evaluate whether combining gemcitabine-carboplatin with adoptive T cell therapy (GC-CTL) improves clinical outcomes for patients with metastatic or locally recurrent EBV-positive NPC	GC-CTL therapy achieved a 71.4% response rate, with 3 CR and 22 PR. At a median follow-up of 29.9 months, the 2-year and 3-year OS rates were 62.9% and 37.1%, respectively. Five patients did not need additional chemotherapy for over 34 months after starting CTL therapy.	–	(83)
	LMP1 LMP2 EBNA1	NCT01462903	I	Unknown status	To investigate the safety and tolerability of autologous TIL in combination with IL-2 following CCRT in advanced NPC patients	Most adverse events were grade 1 or 2. Nineteen out of 20 patients showed an objective antitumour response, with 18 achieving DFS for over 12 months. The plasma EBV load significantly decreased after one week of ACT and stayed below measurable levels in most patients after 6 months of ACT.	The outcome and efficacy of TIL immunotherapy was not clearly defined in the current study.	(84)
	LMP2 EBNA1	NCT00834093	II	Completed	To evaluate the efficacy of EBV-CTLs for recurrent, metastatic NPC patients	Few severe adverse events were observed. The ORR was 4.8%, with a median PFS of 2.2 months. Notably, two patients who had previously failed the same chemotherapy regimen responded well after EBV-CTL immunotherapy.	The number of evaluable participants did not meet protocol objectives (n=13 versus target n=18).	(85)
		Case report			To evaluate the efficacy of combining EBV-CTL with PD-1 blockade therapy in treating metastatic NPC patient	The patient showed complete resolution of metastatic disease with no evidence of disease relapse for 22 months following combination immunotherapy. Subsequent immunological analysis showed a significant change in the overall variety and composition of the T cells, which was linked to the observed clinical improvement.	The findings from this single case may not be directly translatable to a larger cohort of NPC patients.	(86)
	LMP1 LMP2 BARF1 EBNA1	NCT02065362	I	Active, not recruiting	To determine the safety of escalating doses of intravenous infusions of autologous TGF-β-resistant EBV-CTL with lymphodepleting chemotherapy in EBV-positive NPC patients	–	–	–
NK		Case report			To assess the efficacy of an allogeneic UCB-NK cell product in an advanced NPC patient after failure of CCRT	Patient was well tolerated. Intracranial metastases did not decrease 10 months after the NK cell treatment, but they decreased significantly at 31 months after the treatment and partially disappeared. The tumour response indicated a PR. Furthermore, all of the intracranial metastases continued to decrease at about 42 months after treatment.	The optimal dose of NK cells was not clearly determined. Further well-designed and randomized studies with larger numbers of patients are needed to fully evaluate this strategy.	(87)
	EGFR	NCT02507154	I/II	Unknown status	To evaluate the safety and efficacy of expanded activated autologous NK cells administered after cetuximab in patients with EGFR-positive, recurrent/metastatic NPC	Combination therapy was well tolerated. All three patients who received twice infusion of NK cells had a relatively long time to disease progression (12 months, 13 months, and 19 months)	While three out of seven patients demonstrated durable stable disease, the overall response was limited, with three subjects experiencing disease progression. Further research with larger cohorts is necessary to validate these findings.	(88)

CAR, Chimeric antigen receptor; EpCAM, Epithelial cell adhesion molecule; PR, Partial response; PFS, Progression free survival; CRS, Cytokine release syndrome; LMP, Latent membrane protein; TCR, T cell receptor; EBV, Epstein-Barr virus; IL-12, Interleukin-12; EBNA1, Epstein-Barr virus nuclear antigen 1; EBV-CTL, EBV-specific cytotoxic T lymphocyte; SD, Stable disease; OS, Overall survival; CR, Complete response; CCRT, Concurrent chemoradiotherapy; DFS, Disease free survival; ACT, Adoptive cell therapy; TIL, Tumour infiltrating lymphocytes; ORR, Overall response rate; BARF1, BamHI-A rightward frame 1; TGF-β, Transforming growth factor-β; NK, Natural killer; UCB-NK, Umbilical cord blood-derived NK cells; EGFR, Epidermal growth factor receptor.

### 3.1 Chimeric antigen receptors T cell

Chimeric antigen receptor (CAR)-T cells are a type of immunotherapy involving the genetic modification of a patient’s T cells to express a synthetic receptor known as CAR, which includes an antigen-recognition domain (often an antibody single-chain variable fragment, scFv), and an intracellular signalling domain (typically CD3ζ) (89). These CAR-T cells can selectively target tumour associated antigen (TAA) via scFv recognition domain, leading to tumour cell elimination through the production of inflammatory cytokines and cytolytic effectors, achieving a long-term potent antitumour activity (89).

**Figure 1:**
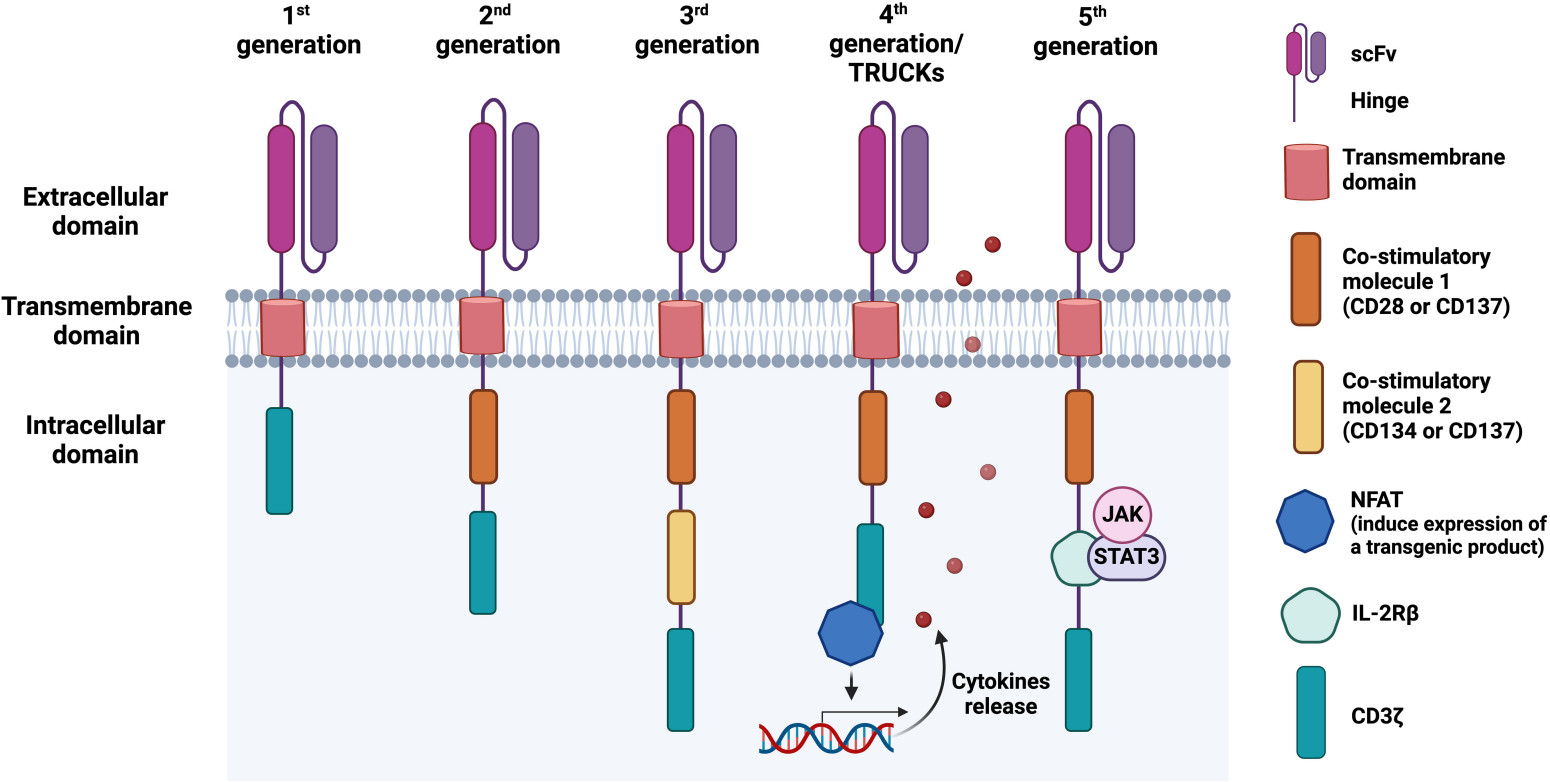
Chimeric antigen receptor (CAR) structure and its evolution from first to fifth generation. The first-generation of CARs consists of an antigen-binding domain, typically a single-chain variable fragment (scFv), followed by a hinge, a transmembrane domain, and an intracellular region, commonly the T cell receptor (TCR) signalling component CD3ζ. In the second and third CAR generations, one or two co-stimulatory domains are added, respectively, enhancing T cell activation and proliferation. Fourth-generation CARs, also known as T cell redirected for universal cytokine-mediated killing (TRUCKs), combine a second-generation CAR construct with additional functional elements, such as cytokine secretion modules. These modules enable CAR-engineered cells to secrete cytokines such as IL-12, which recruit immune cells to the tumour microenvironment (TME) upon antigen recognition, enhancing antitumour activity. The fifth-generation CAR-T cells, also referred to as the next generation, contain a truncated IL-2 receptor β (IL-2Rβ)-chain domain with a motif for binding transcription factors such as STAT3. This can lead to JAK/STAT activation and subsequent cytotoxic responses.

CAR-T cell development has evolved over generations with improved efficacy (**Figure 1**). The first-generation CARs were developed in 1993 by Zelig Eshhar and consisted of a single signalling domain derived from the CD3ζ chain of the TCR (90). However, due to a low cytotoxicity, proliferation rate, and relatively short persistence in patients, the second-generation CARs were developed with an additional co-stimulatory domain (4-1BB/CD137 or CD28), resulting in enhanced T cell proliferation, cytotoxicity, and sustained response (91, 92). Soon, the third-generation CARs are further improved by including additional co-stimulatory domain, such as OX40/CD134 or CD137 (93, 94). CD28 and CD134 support the long-term T cell expansion and survival (95, 96), while CD137 to enhances T cell proliferation, IL-2 production, expression of anti-apoptotic genes to inhibit activation-induced cell death (AICD), and memory T cell development (97). Due to the undesirable clinical outcome, fourth-generation CARs, also known as T cell redirected for universal cytokine-mediated killing (TRUCKs) incorporated a nuclear factor of the activated T-cell (NFAT)-responsive promoter into the base of the second-generation CARs. This promoter can induce cytokine secretion, such as IL-7, IL-12, IL-15, and IL-18 upon CAR cell activation, augmenting T cell activation and killing of cancerous cells (98, 99). In the fifth-generation CARs, a truncated IL-2 receptor β (IL-2Rβ)-chain domain is introduced to provide a binding site for the transcription factor STAT3 and to activate JAK-STAT signalling pathway, further improving T cell activation, proliferation, and persistence (100).

In recent years, CAR T-cell therapy has demonstrated remarkable success in treating relapsed or refractory haematological malignancies. As of January 2024, the FDA has approved six CAR-T cell products: Abecma and Carvykti for multiple myeloma, Breyanzi and Yescarta for large B-cell lymphoma, Kymriah for non-Hodgkin lymphomas, and Tecartus for B-cell acute lymphoblastic leukaemia. Four target CD19, while Abecma and Carvykti target B cell maturation antigen (BCMA) (11). Despite the success in haematological malignancies, CAR-T cell therapies have not received clinical approval for the treatment of solid tumours, including NPC, due to challenges such as, the lack of specific antigens, on-target off-tumour effects, and the complexity of the TME (11). Encouragingly, clinical trials are ongoing. A Phase 1 clinical trial (NCT02915445) targeting epithelial cell adhesion molecule (EpCAM) in solid tumours, including NPC, showed promising antitumour efficacy and an acceptable safety profile, with two patients showing a partial response and three experiencing more than 23 months of progression-free survival (PFS) (80). Engineered T cells targeting LMP1 in EBV-positive NPC cells have also demonstrated increased IFN-γ and IL-2 production, effectively killing LMP1-overexpressing NPC cells *in vitro* and reducing tumour growth *in vivo* (73, 74). The safety and efficacy of these CAR-T cells have been evaluated in a Phase I/II clinical trial (NCT02980315) for the treatment of EBV-associated NPC. Additionally, an ongoing early phase I study (NCT04657965) is assessing these engineered T cells in patients with LMP1-positive infectious diseases and haematological malignancies.

**Figure 2:**
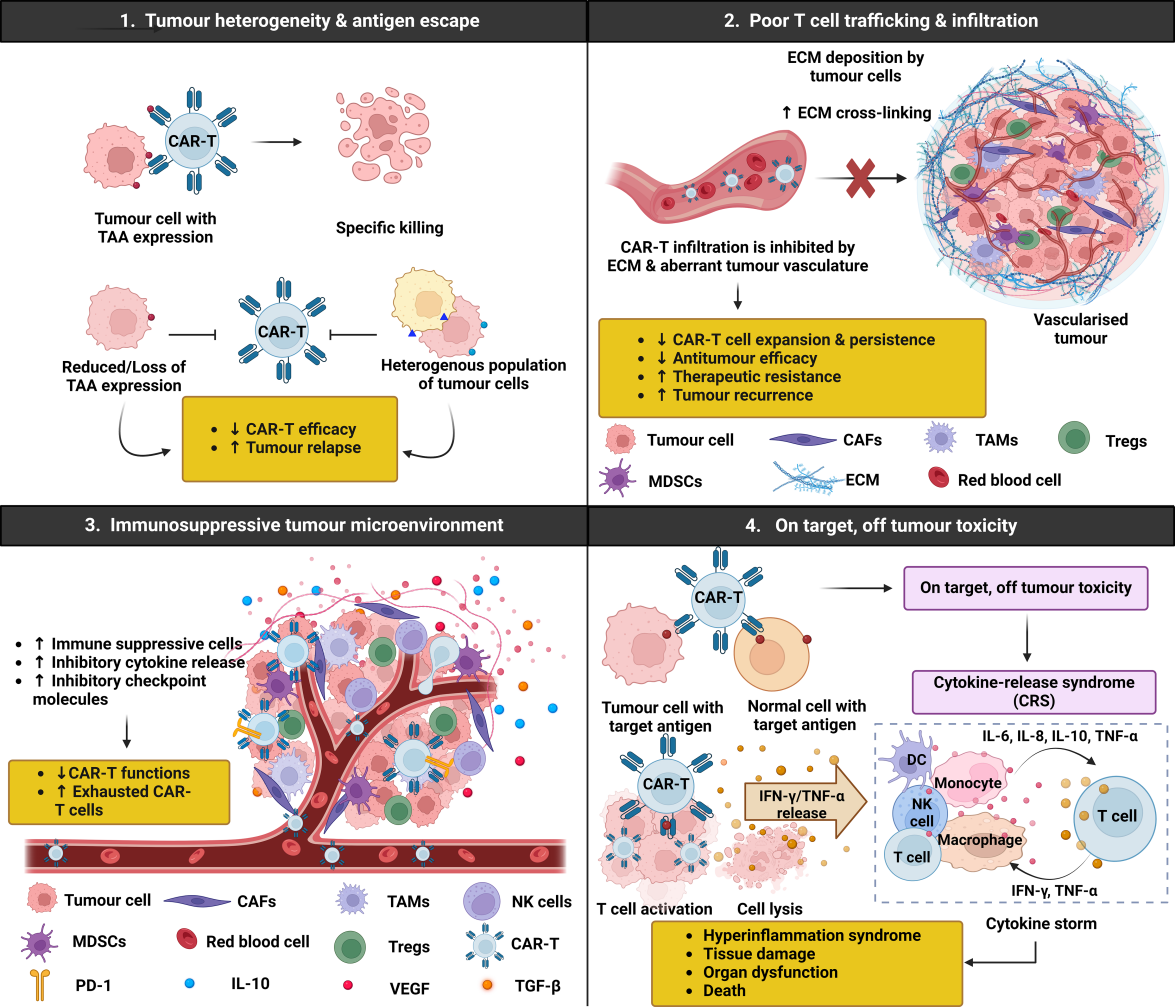
Chimeric antigen receptor (CAR)-T challenges in solid tumours. CAR-T cell immunotherapy has emerged as a promising therapeutic strategy for haematological malignancies, but its application in solid tumours presents several challenges and limitations that need to be addressed. Firstly, the heterogenous nature of tumour cells results in various genetic mutations and antigen expression patterns, making it difficult for CAR-T cells to effectively target all tumour cells. Additionally, tumour cells may undergo antigen escape, where they downregulate or lose the target antigen recognised by the CAR-T cells, allowing them to evade immune detection and destruction. Secondly, the clinical efficacy of CAR-T cell therapy is limited by the inability of CAR-T cells to traffic and infiltrate the tumour due to increased extracellular matrix (ECM) density and abnormal vasculature, ultimately hindering CAR-T cell diffusion and expansion. Thirdly, solid tumours often harbour a complex and highly immunosuppressive microenvironment, which can compromise the effectiveness of CAR-T cell therapy by dampening the immune response and promoting tumour growth. Moreover, higher surface expression of immune checkpoint molecules such as programmed death 1 (PD-1) on CAR-T cells can induce an exhausted phenotype, characterised by reduced cytokine production, proliferation, and cytotoxicity, limiting their capacity to control tumour growth. Lastly, CAR-T cells targeting tumour cells can induce the release of cytokines such as interferon (IFN)-γ and tumour necrosis factor (TNF)-α, activating other immune cells including dendritic cells (DCs), natural killer (NK) cells, monocytes, macrophages, and T cells. These cells further release pro-inflammatory cytokines, triggering a cascade reaction and ultimately contributing to the onset of cytokine release syndrome (CRS), a potentially severe and systemic inflammatory response.

It is also worth noting that numerous challenges limit the therapeutic efficacy of CAR-T cells in both hematological malignancies and solid tumours (**Figure 2**). These challenges include antigen escape, poor tumour infiltration, low persistence, and the immunosuppressive nature of the TME (101). On-target off-tumour recognition is also a concern as many solid tumour antigens are expressed in normal tissues at varying levels. For example, HER2 CAR-T cells may induce severe side effects, including respiratory distress, cardiac arrest, and cytokine-release syndrome (CRS)-induced multi-organ failure. This is because HER2 is expressed not only by malignant cells, but also on normal epithelial cells in various tissues, making them off-targets by HER2 CAR-T cells (102). Therefore, selecting a suitable antigen for CAR-T cell therapy and enhancing the selective expression of CARs for targeting solid tumours are crucial considerations in current research. High rates of severe adverse events with fatal outcomes have prevented CAR-T cells from becoming first-line treatment. In a Phase I clinical trial (NCT01044069) for relapse B-cell ALL patients who received CD19-specific CAR T cells, potent antitumour efficacy of CAR-T cells was observed. However, 14 out of 53 patients experienced severe CRS, and tragically, one patient died from it (103). Neurotoxicity is also common after CAR-T cell therapy, with reports in 33 out of 53 patients, 22 of whom developed severe neurotoxicity within 28 days of CAR-T cell infusion (104). These challenges highlight the need for ongoing improvements and alternative approaches such as engineered TCR-T cells.

### 3.2 Engineered T cell receptor-T cell therapy

The TCR is a crucial component on the T cell surface for recognising antigens on infected or tumour cells. It consists of two antigen-binding peptide chains and three CD3 subunits (ζζ homodimers and δϵ and γϵ heterodimers), forming an octamer complex. Most T cells have αβ TCRs, while a smaller subset has γδ TCRs. The TCR chains comprises an extracellular region for antigen recognition, a transmembrane region, and a shorter cytoplasmic tail linked to the CD3 complex for signal transduction (105). However, due to their lack of co-stimulatory functions, TCRs often require additional co-stimulatory signals for effective T cell activation.

Similar to CAR-T cell therapy, TCR-T cell therapy involves genetically engineering T cells with *TCR* genes to specifically target tumour-specific antigens (TSAs) or TAAs. Unlike CAR-T cells, which recognise antigens directly in an MHC-independent manner, TCR-T cells use naturally occurring TCRs that recognise peptides presented by MHC molecules on the cell surface (106). This involves the proteasomal degradation of cellular proteins, transport into the endoplasmic reticulum (ER), and complex formation with MHC molecules. The peptide-MHC complexes are then presented on the cell surface for T cell surveillance (107). It has been reported that TCR-T cells exhibit nearly 100- to 1000-fold greater sensitivity in recognising peptide-HLA complexes compared to CAR-T cells, enhancing immune recognition and the tumour elimination (108, 109). This high specificity leads to more selective and regulated T cell activation, reducing the risk of excessive activation and cytokine production, thus resulting in milder treatment-related toxicity compared to CAR-T cell therapy (110, 111). Therefore, TCR-T cells offer an enhanced therapeutic strategy for targeting a broader range of malignancies compared to CAR-T cell therapy.

Over the past decade, therapies involving the EBV-specific T cell infusion have shown remarkable clinical responses in treating post-transplant lymphoproliferative disease (PTLD), a life-threatening complication following organ transplantation (112). Subsequent studies have explored the use of TCRs specific to EBV viral antigens, such as EBNAs and LMPs, in treating EBV-associated malignancies, including NPC. For example, retroviral transduction of engineered EBV antigen-specific TCRs into primary human T cells have demonstrated increased IFN-γ production and efficient lysis of tumour cells (113). Cho and co-workers developed a TCR specific to LMP1 from HLA-A*0201 transgenic mice immunised with the minimal epitope LMP1_166_ (TLLVDLLWL). Infusion of these engineered T cells into immunocompromised mice revealed specific activation by low peptide concentrations and efficient recognition of LMP1-expressing tumour cells, demonstrating high avidity for antigen recognition in EBV-associated malignancies (23). Promising outcomes were also observed with LMP2-specific TCR, where transduced CTLs increased IFN-γ production and specific lysis of target cells. Infusion of these CTLs significantly attenuated the growth of LMP2-expressing tumours *in vivo*, improving survival rates in tumour-bearing mice (75). Additionally, transgenic T cells expressing LMP2-specific TCRs showed high avidity antigen-specific functions, including proliferation, cytotoxicity, and cytokine release (IFN-γ, TNF-α, and IL-2) against EBV-positive NPC cells. These engineered T cells effectively suppressed LMP2-positive tumour growth in an immunocompromised mouse model and specifically recognised and targeted LMP2-expressing NPC cells in advanced NPC patients (76). Taken together, these findings highlight the potential of TCR-T cell therapies in redirecting T cells to recognise EBV antigens, demonstrating efficacy in combating EBV-associated malignancies such as NPC.

At present, TCR-T cell therapies have not received FDA approval for clinical application in solid tumours other than melanoma. However, extensive efforts are ongoing to evaluate their safety and efficacy in other cancers, including NPC. TCR-T cells have also been developed to target cancer-associated viral antigens, such as EBV, HPV, and hepatitis B virus (HBV), and are being tested in multiple clinical trials. In a phase I/II clinical trial (NCT02280811) with HPV16-E6-specific TCR-T cells, two of 12 chemotherapy-refractory, metastatic HPV16-positive cancer patients experienced objective tumour responses without off-target toxicities or dose-limiting toxicities (114). In another phase I clinical trial (NCT03899415), HBV-targeted TCR T cells for HBV-related advanced HCC post-hepatectomy or radiofrequency ablation showed promising results. Among the eight patients, one achieved a partial response lasting 27.7 months, with only mild adverse events reported. Additionally, circulating HBV antigens and HBV DNA loads were reduced in all patients after TCR-T cell infusions, indicating effective targeting (115). For NPC, multiple clinical trials are focusing on TCR-T cells targeting EBV viral antigen such as LMP1 and LMP2. An ongoing phase I/II study (NCT05587543) is investigating LMP2-specific IL-12-secreting TCR-T cells in EBV-positive metastatic/refractory NPC patients. Another phase I trial (NCT03925896) is evaluating the safety and efficacy of LMP2-specific TCR T cells for HLA-A2, HLA-A11, and HLA-A24 recurrent and metastatic NPC patients. Additionally, a phase II study (NCT03648697) is assessing the safety and tolerability of LMP1, LMP2 and EBNA1-specific TCRs (YT-E001) in HLA-A02:01-/24:02-/11:01-positive recurrent or metastatic NPC patients. These high affinity TCRs targeting specific EBV antigens were screened from healthy donors and transduced into autologous T cells via lentiviral vectors.

Despite their promise, TCR-T cell therapies face challenges, including antigen selection, tumour heterogeneity, T cell exhaustion, and safety concerns. Ideal target antigen should be selectively and homogeneously expressed in tumours and presented via MHC class I to minimise off-target effects and treatment-related toxicity. However, solid tumours often exhibit antigenic variability, increasing the risk of antigen escape and the emergence of TCR-T cell-resistant tumours (116, 117). The immunosuppressive TME further impedes TCR-T cell infiltration and their efficacy in eliminating tumour cells (118). Additionally, prolonged TCR stimulation can induce T cell exhaustion, characterised by upregulated immune checkpoint proteins, such as PD-1, CTLA-4, T cell immunoglobulin and mucin domain-containing protein (TIM)-3, and LAG-3 (119). Tumours can also evade TCR-T cell recognition through mechanisms, such as downregulation of loss of MHC class I molecules through genetic alterations or epigenetic silencing (120). For example, non-responsive patients in a phase I/II clinical trial (NCT02858310) targeting HPV-16 E7 antigen showed tumour resistance due to defects in antigen presentation and interferon response pathways (121). Therefore, developing screening assays to identify genomic alterations associated with treatment resistance is important in order to improve TCR-T cell therapy efficacy.

### 3.3 Tumour-infiltrating lymphocytes therapy

Tumour-infiltrating lymphocytes (TILs) represent a subset of intratumour lymphocytes, and their adoption for treating advanced solid tumours has shown promising clinical outcomes. TIL therapy involves isolating these cells from the tumour, cultivating them *in vitro*, and reinfusing them into the patient with a high dose of IL-2 after lymphodepletion to enhance T cell survival and target tumour cells (122, 123). The efficacy of TIL therapy was first established by Steven Rosenberg in 1988 (124), and has now shown encouraging outcomes in NPC.

EBV antigens in NPC have led to the exploration of EBV-specific T cells as an alternative treatment. Studies showed that TILs isolated from NPC patients consists of a high frequency of CD4^+^ T cells that produce IFN-γ in response to EBNA1, aiding tumour regression (125). A number of clinical studies have delved into assessing the effectiveness of EBV-specific T cells in patients with NPC. These investigations have revealed promising trends, notably in the alteration of EBV DNA copy numbers and the augmentation of CTL levels, indicating a potential therapeutic benefit of EBV-specific T cell therapy in NPC patients. For instance, a phase I clinical study targeting EBV-positive NPC resistant to RT and chemotherapy with EBV-specific CTLs showed acceptable safety and modest objective responses. Among the 10 participants, two displayed partial responses and four maintained stable disease states (81). Building on this success, a subsequent phase II study in 23 recurrent or refractory NPC patients showed well-tolerated autologous EBV-CTL infusion, with 1-year and 2-year PFS rates of 65% and 52%, and overall survival rates of 87% and 70%, respectively (82). Furthermore, in a phase III clinical trial (NCT02578641), when EBV-CTLs were used to treat NPC patients after completing a first course of chemotherapy, the response rate increased to 71.4%, including three complete responses and 22 partial responses (83). A phase I study (NCT01462903) evaluating the safety and antitumour activity of TILs following CCRT in locoregionally advanced NPC (LANPC) patients showed promising results, including sustained antitumour activity, extended disease-free survival (> 12 months in 18 out of 20 patients), and enhanced EBV antigen-specific T cell responses (84). However, a completed phase II study (NCT00834093) evaluating EBV-specific CTLs in patients with recurrent, metastatic NPC demonstrated a poor overall response rate (ORR) and a median progression-free survival (PFS) of only 2.2 months. Of the 28 patients enrolled, 21 were treated, only one patient achieved a complete response, two experienced stable disease, and the remaining patients had disease progression. Interestingly, two patients who had previously failed the same chemotherapy regimen showed a renewed and robust response to chemotherapy after receiving EBV-CTL immunotherapy (85). These findings suggest that the efficacy of EBV-CTL immunotherapy may be enhanced when used in combination with other conventional therapies. Combination therapies, such as EBV-specific CTL with PD-1 blockade, have shown potential, as evidenced by a complete response with no evidence of disease relapse for 22 months in a metastatic EBV-positive NPC patient, suggesting potential for synergistic combination therapies (86). An ongoing phase I clinical trial (NCT02065362) takes a step further by examining the antitumour activity of TGF-β resistant, EBV-specific CTLs in patients with EBV-positive NPC. These genetically modified T cells incorporate a dominant negative receptor (DNR), conferring resistance to TGF-β and augmenting their efficacy in eliminating tumour cells. The ongoing or completed clinical approaches utilising EBV-specific TILs for treating NPC are summarised in **Table 4**.

**Table 4:** Clinical trials of EBV-specific TIL therapy in NPC. .

Inter-vention	Cell source	Preparation process	Disease	Phase	Infusion protocols	Findings	Clinical trial identifier/ Reference
EBV-CTL alone	PB	1. Isolate PBMCs. 2. Incubate with EBV-containing supernatant from the B95-8 cell line to establish LCL in the presence of cyclosporine-A. 3. Expand CTLs by weekly stimulations with LCLs in the presence of recombinant IL-2	Stage IV radiotherapy- and chemotherapy- resistant EBV-related NPC	I/II	Autologous CTLs were infused weekly for the first four administrations and then every 2 to 4 weeks, with low-dose recombinant IL-2. The first five patients received an initial dose of 2 x 10^7^ CTL/m^2^ followed by subsequent doses of 4 x 10^7^ CTL/m^2^. For the last five patients (patients 6 to 10), the schedule was modified to include four escalating doses of EBV CTL (2 x 10^7^, 4 x 10^7^, 6 x 10^7^, and 8 x 10^7^ CTL/m^2^) every 2 weeks, followed by infusions of 6 x 10^7^ CTL/m^2^.	• Induced LMP2-specific immunologic responses • Well tolerated with no reported acute adverse effects • 4 out of 10 patients achieved stable disease, lasting a median of 6 months	(81)
EBV-CTL alone	PB	1. Isolate PBMCs. 2. Incubate with EBV-containing supernatant from the B95-8 cell line to establish LCL in the presence of cyclosporine-A. 3 .Expand CTLs by weekly stimulations with LCLs in the presence of recombinant IL-2.	Relapsed/ refractory EBV-positive NPC	I/II	Patients were treated at one of three dose levels and received either 2 doses of 2×10^7^ CTL/m^2^ (dose level 1), 1 dose of 2×10^7^ and 1 dose of 1×10^8^ CTL/m^2^ (dose level 2), or 1 dose of 1×10^8^ and 1 dose of 2×10^8^ CTL/m^2^ (dose level 3). All patients in the Phase II extension received the highest dose level. CTL infusions were given 2 weeks apart.	• No significant toxicity was reported • Demonstrated progression-free survival rates of 65% at first year and 52% at second year • Achieved overall survival rates of 87% at first year and 70% at second year	(82)
EBV-CTL + Gemcitabine + Carboplatin	PB	1. Isolate PBMCs. 2 .Incubate with EBV-containing supernatant from the B95-8 cell line to establish LCL in the presence of cyclosporine-A. 3. Expands CTLs by weekly stimulations with LCLs in the presence of recombinant IL-2.	Metastatic and/or locally recurrent NPC	II	Patients received chemotherapy with gemcitabine (1,000 mg/m^2^) and carboplatin (AUC 2) on days 1, 8, and 15 every 4 weeks for four cycles. Two to four weeks after the last chemotherapy course, EBV-CTLs were administered at a dose of 1 × 10^8^ cells/m^2^ on weeks 0, 2, 8, 16, 24, and 32.	• Well tolerated with no grade 3 or 4 adverse events • Achieved a response rate of 71.4% • Out of 35 patients, 3 achieved a complete response, 22 had a partial response, and 10 had stable disease • Demonstrated 1-, 2-, and 3-year overall survival rates of 77.1%, 62.9%, and 37.1%	NCT02578641 (83),
EBV-TILs + CCRT	Biopsied NPC tumours	1. Mince NPC biopsy specimens into to isolate TILs. 2. Digest with collagenase type IV (0.1 μg/mL) for 2 hours. 3. Culture in X-VIVO-15 medium with 5% human AB serum and recombinant IL-2.	Locoregio-nally advanced NPC	I	The PTV of the GTVnx was treated with 70 Gy in 30–32 fractions. Cisplatin was administered at 100 mg/m^2^ on days 1, 22, and 43 of radiotherapy. One week after completing CCRT, patients received a single-dose intravenous infusion of TILs (average infused TIL number = 2.6 × 10^9^ ± 2.2) and began a 14-d regimen of low-dose IL-2 subcutaneous injection.	• Induced EBV-specific T cell expansion in 13 of 20 patients • No treatment-related deaths. Only mild adverse events were reported • 18 out of 20 patients exhibited disease-free survival longer than 12 months • Plasma EBV load was undetectable in 17 patients at 6 months after this therapy	NCT01462903 (84),
EBV-CTLs	PB	1. Isolate PBMCs. 2. Incubate with EBV-containing supernatant from the B95-8 cell line to establish LCL in the presence of cyclosporine-A. 3. Expand CTLs by weekly stimulations with LCLs in the presence of recombinant IL-2.	Recurrent, metastatic NPC	I/II	Each subject received two EBV-CTL infusions, given 2 weeks apart at a dose of 1×10^8^ cells/m². Eight weeks after the second infusion, subjects underwent restaging scans to evaluate their response using RECIST (version 1.1). If additional product was available, patients could receive one more infusion (1-2×10^8^ cells/m²) approximately 8 to 12 weeks after the second infusion.	• One out of 21 treated patients achieved complete response • Two patients maintained stable disease for 18.7 months and 6.5 months • Overall response rate was 4.8% • Median progression-free survival was 2.2 months • Median overall survival was 16.7 months • No grade ≥ 3 adverse events were found to be related to treatment	NCT00834093 (85),
EBV-CTLs + Nivolumab	PB	1. Isolate PBMCs. 2. Coculture with irradiated PBMCs infected with AdE1-LMPpoly (MOI 10:1) at a responder to stimulator ratio of 7:3. 3. On Day 3, and every 3 to 4 days, add recombinant IL-2 to the medium. 4. Harvest the cells on Day 14.	EBV-positive metastatic NPC	I/II	The patient was treated with nivolumab at 240 mg per cycle (14 days). Following this, the patient received six intravenous infusions of *in vitro*-expanded T cells at a dose of 4 × 10^7^ T cells (2 × 10^7^ cells/m^2^).	• EBV DNA remained undetectable for more than 250 days after the completion of combination therapy • No signs of disease recurrence for 22 months • EBV-specific CTLs in PBMC increased from 0.16% to 0.33% following the ACT product	(86)
TGF-β resistant EBV-CTL	PB	1. Isolate APCs from the patient’s blood. 2. Coculture with K562 cells coated with a mixture of LMP, EBNA1, and BARF protein fragments to generate EBV-specific T cells.	EBV-positive NPC	I	Patients will be treated with (1) either two doses of EBV-specific T cells, with the second dose administered 2 weeks after the first dose or (2) cyclophosphamide and fludarabine for 3 days before receiving the EBV-specific T cells.	• Ongoing clinical study • No clinical data is available	NCT02065362

EBV, Epstein-Barr virus, TIL, Tumour-infiltrating lymphocytes; NPC, Nasopharyngeal carcinoma; CTL, Cytotoxic T lymphocytes; PB, Peripheral blood; PBMC, Peripheral blood mononuclear cells; LCL, B-lymphoblastoid cell line; IL-2, Interleukin-2; LMP2, Latent membrane protein 2; CCRT, Concurrent chemoradiotherapy; PTV, Planning target volumes; GTVnx, Gross tumor volume in the nasopharynx; RECIST, Response Evaluation Criteria in Solid Tumours; MOI, Multiplicity of infection; ACT, Adoptive T-cell therapy; TGF-β, Transforming growth factor-beta; APCs, Antigen presenting cells; EBNA1, Epstein-Barr nuclear antigen 1; BARF, BamHI A right frame.

Despite promising outcomes, TIL-based therapy encounters significant challenges. Initial steps involve invasive surgical resection for TIL isolation can be distressing and poses risks to patients (126). The heterogeneity of TILs in terms of antigen specificity and differentiation stages may affects their effectiveness against tumours, impacting the overall success of the therapy (127). Challenges in TIL expansion and preparation include the need for specialised facilities and technical expertise for TIL culture and expansion (128), and patient factors such as age and overall health may influence the success of *ex vivo* expansion (129). Furthermore, the lengthy process of isolation, expansion, and reintroduction into the patients’ body is around 3 weeks to 3 months, considered a prolonged duration that inevitably generates delays for patient intervention (130). Ongoing research aims to optimise expansion protocols and shorten production times, while exploring combination therapies involving TILs and conventional treatments that hold potential for synergistic efficacy in cancer therapy.

### 3.4 Natural killer cell therapy

Natural killer (NK) cells offer a promising alternative for cellular immunotherapy beyond T cells. As a key component of the innate immune system, NK cells play a vital role in cancer immune surveillance, eradicating tumour cells in an antigen-independent manner without requiring HLA matching (131). Their cytotoxic effects involve releasing perforin and granzymes, and activating killer activating receptors (KARs), leading to apoptosis via the expression of death ligands, such as TNF-α, FasL, and TRAIL. NK cells also modulate immunity by producing cytokines and chemokines, including IFN-γ, IL-10, CCL3, CCL4, and CCL5 (132). Several studies have confirmed the close relationship between NK cells and cancer development, with higher NK cell activity correlating with reducing susceptibility to oncogenic virus infection and improved survival (133, 134).

Adoptive transfer of autologous NK cells for cancer treatment is feasible due to easy sourcing and low risk of GVHD, but its limited tumour regression restricts clinical application (135). Researchers now focus on allogeneic NK cells, which offer advantages such as MHC-unrestricted immune recognition and lower GVHD incidence compared to CAR-T cell therapy. These cells, derived from PB, UCB, hESCs, iPSCs, or NK-92 cell lines, provide versatility for large-scale manufacturing and cryopreservation, allowing off-the-shelf availability (131). Among these, PB is the most accessible source but has limitations such as low cell counts (136), reduced proliferation and short lifespan (137), decreased cytotoxicity after cryopreservation (138), donor variability (139), and heterogenous NK cell populations, may potentially lead to variable NK cell function (140).

In contrast, UCB-derived NK cells offer several advantages over PB, including easier collection and long-term cryopreservation (141), better proliferation (142), enhanced bone marrow homing ability (143), and lower GVHD incidence (144). However, they have higher CD161 expression, limiting maturity and response to foreign antigens (143). The lower expression of adhesion molecules such as intercellular adhesion molecule (ICAM)-1 also limit their capacity to form conjugates with target cells (142, 145). They also exhibit higher expression of inhibitory molecules such as NKG2A/CD94, lower expression of CD16 (receptor that facilitates ADCC), CD57 (NK cell maturation marker), and KIR2DL4 (activating receptor), and reduced production of perforin, granzyme B, IFN-γ, and cell surface FasL and TRAIL, reducing cytotoxicity against tumour cells (146). However, cytokines such as IL-2, IL-12, IL-15, and IL-18 can restore UCB-NK cell cytotoxicity (147), and monoclonal antibodies such as monalizumab can enhance NK cell activity by inhibit the inhibitory function of NKG2A (148).

As discussed earlier, hESCs/iPSCs can differentiate into NK cells, offering an alternative source for allogeneic NK cells by providing homogenous cell populations that can be grown indefinitely. Their potential for genetic engineering with CARs makes them a promising strategy to generate standardised, off-the-shelf NK cells with enhanced expansion, *in vivo* persistence, and improved antitumour activity (149, 150). Immortalised NK cell lines such as NK-92, which can be expanded *ex vivo*, are also promising (151, 152). NK-92 shows high antitumour activity and are the only cell line approved for CAR-NK-92 clinical trials (153). It was established in 1992 from a non-Hodgkin lymphoma patient, showing characteristics of early NK cells with the expression of CD56, CD2, and CD7, but lack CD3 (154). The continuous growth of NK-92 is IL-2 dependent, and despite lacking CD16, they exhibit significant cytotoxicity due to the expression of activating receptors, including NKp30, NKp46, NKG2D, and CD28, with almost complete loss of inhibitory killer cell immunoglobulin-like receptors (KIRs) on the surface, except for KIR2DL4. NK-92 cells also express high levels of cytotoxic effector molecules, such as perforin, granzyme B, FasL, TRAIL, and TNF-α, consistently inducing high cytotoxic activity against tumour cells (155). In order to minimise the risk of developing lymphoma in recipients due to the origin of NK-92 cells, they require irradiation before infusion to prevent continuous growth while maintaining their cytotoxicity (156). Multiple phase I trials (NCT00990717, NCT00900809) have evaluated the safety and efficacy of irradiated NK-92 cells, showing favourable clinical outcomes, further reinforcing the potential of their therapeutic application (157, 158). However, irradiation may limit their expansion capacity, potentially diminishing overall antitumour efficacy (159).

There is growing research on NK cell-based therapy for NPC. LMP2 antigens are potential targets for NK cells, with targeted clearance of LMP2-containing cells showing robust antitumour effects and minimal toxicity to normal cells. During EBV infection, LMP2A induces F3 expression via the PI3K/Akt signalling pathway, negatively affecting NK cell activation (77). F3 promotes platelet aggregation, potentially aiding cancer metastasis and evading NK cell surveillance by downregulating NK2GD ligands and suppressing IFN-γ release (160). Additionally, platelet-derived TGF-β further enhances tumour metastasis by inhibiting the expression of CD226 and CD96 on NK cell surface, protecting tumour cells from being recognised by NK cells (161). Inhibition of F3 with the administration of NK-92MI (independent of exogenous IL-2) or UCB-derived NK cells has been shown to restore NK cell antitumour function in an NPC xenograft mouse model (77). UCB-derived NK cells also suppressed brain metastasis in a recurrent NPC patient post chemoradiotherapy, showing significant reduction over two years, with sustained efficacy (87). Combining RT with PD-1 inhibition synergistically increases NK cell-mediated killing of NPC cells *in vitro* and *in vivo* (78). Additionally, LMP1 induced B7-H3 expression via PI3K/AKT/mTOR pathway activation, leading to a reduction of NK cell cytotoxicity. Deleting B7-H3 in tumour cells and using anti-PD-L1 treatment restored NK cell function and enhanced cytotoxicity against NPC cells in xenograft models, suggesting the potential when combined with NK cell-based immunotherapy and immune checkpoint blockade against EBV-associated NPC (79). In a recent Phase I/II study (NCT02507154), Lim and co-workers assessed the safety and efficacy of combining cetuximab (anti-EGFR) with autologous expanded NK cells in EGFR-positive, recurrent and/or metastatic NPC patients who had failed at least two prior lines of chemotherapy. The treatment was well tolerated, with three out of seven patients achieved stable disease after the first NK cell infusion and experiencing a relatively long time to disease progression, lasting up to 19 months with two NK cell infusions (88).

### 3.5 CAR-NK cell therapy

NK-92 cells, known for their long-term cryopreservation capability and uniform population, are increasingly integrated with cancer-targeting CARs (CAR-NK-92). The first-generation CAR-NK cells feature a single signalling domain (CD3ζ), which is insufficient for potent killing. In contrast, the second- and third-generation CAR-NK cells incorporate additional co-stimulatory molecular motifs, such as CD28 or CD137 (4-1BB) for improved efficacy (162). For instance, anti-CD-19/CD22 bispecific CAR-NK-92 cells incorporating a CD3ζ/CD137 signalling domain exhibited increased cytotoxicity against B cell lymphoma cells compared to anti-CD19 CAR-NK-92 cells alone (163). Third-generation CAR-NK-92 cells targeting HER1 in HNSCC demonstrated enhanced antitumour immune responses characterised by increased INF-γ secretion and CD107a expression, a degranulation marker. Despite their enhanced killing activity, challenges such as PD-L1 upregulation and expansion of CD44v6-positive (putative CSC marker) on surviving HNSCC cells have been reported (164). This suggests that relying solely on CARs targeting a single TAA may not be effective as monotherapy, potentially leading to tumour relapse and treatment resistance. Therefore, combination therapy approaches are necessary to enhance CAR-NK cell efficacy. For instance, second-generation CAR-NK-92 cells co-expressing anti-HER2 and soluble PD-1 significantly increased NK cell and T cell infiltration and effector molecule release, enhancing immunological antitumour efficacy in PD-L1^+^Her2^+^ breast cancer cells (165). Similarly, a third-generation CAR targeting epidermal growth factor receptor (EGFR) in NK-92 cells, combined with the kinase inhibitor cabozantinib, effectively lysed EGFR-positive renal cell carcinoma (RCC) cells and improved tumour homing (166).

Currently, only a few registered clinical trials on the US Clinical Trials Registry (ClinicalTrials.gov) investigate CAR-NK-92 for hematological or solid tumours. For instance, a phase I clinical trial (NCT02944162) assessed the safety profile and clinical efficacy of third-generation CAR-NK-92 cells targeting CD33 in relapsed/refractory AML (167). Although no significant clinical efficacy was observed due to the decreased cytotoxic potential following irradiation, the therapy demonstrated safety with a high cell dose (five billion cells per patient) (167). Another ongoing phase I clinical trial (NCT03383978) is evaluating the safety and tolerability of a second-generation HER-2 specific CAR-NK-92 for recurrent glioblastoma. The trial has shown a favourable safety profile with local intracerebral injections of up to 1 x 10^8^ HER2-CAR-NK-92 cells (168). New advancements include high-affinity Fc-receptor-expressing NK cells (haNKs), derived from NK-92 cells, which can be genetically engineered to express CARs, augmenting their targeting capabilities and cytotoxic potential (169). CAR-haNK cells targeting PD-L1 have demonstrated successful recognition and targeting of heterogeneous tumour cell populations, including both T cell-sensitive and T cell-resistant tumour cells, in an HNSCC mouse model, effectively overcoming immune resistance (170). A phase II clinical trial (NCT04847466) is evaluating the effectiveness of irradiated PD-L1 CAR-haNK cells in combination with the PD-1 inhibitor pembrolizumab plus N-803 (ALT-803, an IL-15 agonist) in patients with recurrent or metastatic gastric cancer and HNSCC. Preliminary studies (NCT04050709) have shown PD-L1 CAR-haNK cells at a dose of 2 x 10^9^ cells twice weekly are well-tolerated, with no dose-limiting toxicities or CRS, supporting their advancement to phase II clinical trial (171). While studies have not yet evaluated CAR-NK products in NPC patients, the promising therapeutic efficacy observed in other solid tumours suggests that exploring genetic engineering of the *ex vivo* expanded NK cells could offer clinical benefits in NPC treatments.

Despite their promise, CAR-NK-92 cells require irradiation before infusion, which affects their viability and cytotoxicity more rapidly than non-irradiated cells (172). Therefore, the dosage and impact of irradiation on CAR-NK-92 cells needs to be carefully considered in future clinical trials. Given the short lifespan of irradiated CAR-NK-92 cells, shortening the interval between infusions may improve therapeutic efficacy. Similar to CAR-T cells, CAR-NK-92 cells exhibit target-dependent cytotoxicity and may induce on-target, off-tumour toxicity if target antigens are expressed in normal tissues (173). To address this, strategies like incorporating suicide genes into CAR constructs can serve as a safety switch, enabling control over engineered cell activity and facilitating elimination if necessary. This approach could improve safety and effectiveness of CAR therapies (174, 175).

## 4 Strategies to improve the efficacy of cell-based therapies in NPC

As discussed earlier, despite the encouraging results of cell-based therapies in hematological malignancies, their application in solid tumours is still in the clinical trial phase and faces numerous challenges. In this section, we will explore several strategies aimed at overcoming the insufficient infiltration of adoptively transferred cell types to the tumour site and improving the efficacy of the cellular therapies.

### 4.1 Alteration of chemokine expression profile

Chemokines are small cytokines that regulate immune cell migration and trafficking. The chemokine expression profile of solid tumours is complex and influenced immune cell recruitment to the TME and tumour growth (176). Strategies targeting chemokine and their receptors are increasingly used in immunotherapy to enhance the effectiveness of CAR-engineered immune cells. For instance, CAR-T cells engineered with IL-7 and CCR2b (7x2b CAR-T) showed improved survival and migration to the tumour site by boosting IFN-γ, IL-2, and granzyme production (177). Similarly, co-expressing CXCR1 in CAR-NK cells targeting NKG2D ligands exhibit better tumour trafficking and growth inhibition (178). Additionally, genetically modified oncolytic viruses (OVs) can alter the tumour chemokine profile, aiding in antigen presentation and boosting chemokine production to recruit immune cells into the TME (179). Studies show that intratumoral administration of a CXCL11-armed tumour selective vaccinia OV increases tumour-specific CTLs and granzyme B production, while reducing immunosuppressive cytokines in the TME of a syngeneic mouse mesothelioma model, leading to enhanced cytotoxic activities of CTLs (180). In another study, Moon and co-workers evaluated the synergistic effects of CXCL11 and mesothelin-redirected CAR-T cells. While CAR/CXCL11 showed limited T cell trafficking, VV.CXCL11, an oncolytic vaccinia virus producing CXCL11, effectively increased T cell infiltration and improved antitumour efficacy after adoptive T cell therapy (181). Thus, combining oncolytic virotherapy with adoptive T cell transfer holds promise for enhancing NPC therapy efficacy.

### 4.2 Targeting extracellular matrix and stromal cells

Solid tumours are enriched with extracellular matrix (ECM), stromal cells, and immunosuppressive cells, creating barriers that hinder immune cell penetration and infiltration (182). To tackle the problem, researchers have engineered CAR-T cells to target ECM components and cancer-associated fibroblasts (CAFs). For instance, CAR-T cells expressing heparanase (HPSE) can break down ECM components and reduce fibrosis, improving immune cell infiltration and antitumour effects (183). Fibroblast activation protein (FAP), a marker distinguishing CAFs from their normal counterparts, has been found in over 90% of epithelial cancers, including NPC, and is often correlated with poor prognosis (184). Preclinical studies shows that CAR-T cells targeting FAP can eliminate CAFs, suppress myeloid-derived suppressor cells (MDSCs) recruitment, and enhance CTL and CAR-T cell survival (185). At present, only two clinical trials have been conducted using anti-FAP CAR-T cells. A phase I clinical trial in malignant pleural mesothelioma (NCT01722149) reported that localized injection of these CAR-T cells was well tolerated with ongoing antitumour immune responses (186, 187). Another phase I clinical trial (NCT03932565) is evaluating the safety of fourth-generation CAR-T cells targeting Nectin4 and FAP, combined with IL-7, CCL19, or IL-12 for advanced Nectin4-positive solid tumours. Pre-clinical studies show that Nectin4-targeted CAR-T cells with IL-7 and CCL19 help prevent CAR-T cell exhaustion by reducing immune checkpoint expression, while FAP-targeted CAR-T cells with IL-12 enhance immune cells recruitment (188). These combination therapies show potential for improving treatment outcomes in NPC.

### 4.3 CAR-T cells secreting bispecific T-cell engagers

Over the past few decades, bispecific antibodies (BsAbs), especially bispecific T cell engagers (BiTEs), have proven effective in treating hematologic malignancies by binding two different antigens to direct immune cells to tumour (189). BiTEs facilitate direct interaction between T cells and tumour cells directly by binding CD3 on T cells and TAA on tumour cells, bypassing the need for antigen-presenting cells (APCs) (190). This approach helps overcome antigen loss and variability, as seen with EGFR variant III (EGFRvIII) CAR-T cells secreting BiTEs to target glioblastoma cells while activating bystander T cells, enhancing the antitumour response against heterogeneous tumours (191). A study by Yin and co-workers showed that BiTE-secreting T cells EGFR and interleukin-13 receptor alpha 2 (IL13Rα2) exhibited superior antitumour activity with higher sensitivity and specificity compared to their CAR-T counterparts in glioblastoma model (192). Overall, these findings suggest that BiTE-secreting CAR-T cells could be a promising approach to address challenges associated with antigen heterogeneity in solid tumours. While research on using BiTEs to enhance CAR-T efficacy in targeting NPC is limited, targeting LMPs expressed on EBV-infected cell surfaces, but not on normal cells, could be explored as a potential approach for BsAbs. This concept is supported by second-generation CAR-T cells targeting LMP1 in LMP1-positive NPC cells, demonstrating specific killing of NPC cells and inhibition of tumour growth in xenograft model (73).

## 5 Strategies to improve the safety of cell-based therapies in NPC

While cell-based therapies represent an innovative treatment in the oncology field, showing promising results in multiple clinical trials, they also carry the risk of potentially life-threatening or even fatal toxicities. In a recent study, Fusaroli and co-workers review post-approval adverse events associated with tisagenlecleucel and axicabtagene ciloleucel between October 2017 and September 2020 using the FDA Adverse Events Reporting System (FAERS) database. This database supports the FDA’s post-marketing safety surveillance program for drug and therapeutic biologic products. The study identified a total of 3225 reports (1793 for axicabtagene ciloleucel and 1433 for tisagenlecleucel), with CRS and neurotoxicity reported as the two major complications. Notably, 75% of these events occurred within the first 10 days of CAR-T therapy (193). Thus, enhancing the safety of cell-based therapies in NPC is critical for their efficacy and clinical application. This section explores strategies aimed at mitigating risks associated with these therapies, ensuring they are both effective and safe for patients.

### 5.1 Improving the safety of CAR-T by DNAX activation protein of 12 kDa (DAP12)

An increasing body of evidence suggests that CAR toxicity may be linked to the synthetic nature of the receptor design (194). To address this, researchers have constructed a natural multi-chain immunoreceptor CAR using DAP12 instead of CD3ζ. DAP12, a 12-kDa transmembrane adaptor protein with a single immunoreceptor tyrosine-based activation motif (ITAM), was originally found to activate NK cells and is involved in transmitting activating signals from various receptors (195). Studies show that DAP12-based CARs offer superior antitumour activity and safety than CD3ζ-based CARs, with improved antigen-specific cytotoxicity, TIL proliferation, reduced toxicity, and lower production of pro-inflammatory cytokines (196, 197), highlighting the potential of DAP12 in mitigating the risk of CRS. For instance, a phase I clinical trial (ChiCTR1800016584) of CD19-KIRS2/DAP12-BB CAR-T cells reported complete responses in all patients with low toxicity (198). Similarly, CAR-NK cells incorporating DAP12 have shown promising results in treating solid tumours. In a recent study, Xiao and co-workers constructed a CAR-NK cell by combining the NKG2D receptor with DAP12, which showed significant therapeutic effects and lower toxicity in mice with solid tumours. This approach also led to positive outcomes in three patients with metastatic colorectal cancer (199). Building on these results, a pilot clinical trial (NCT03415100) has been launched to evaluate the safety and feasibility of these CAR-NK cells for treating metastatic solid tumours.

### 5.2 Incorporation of suicide genes to address the challenge of toxicity

Another strategy to enhance the safety of CAR-based cell therapy is the engineering of suicide genes such as inducible caspase 9 (iCasp9) into the CAR construct. These suicide genes serve as a safety switch that can induce cell death upon activation by an external agent, such as drug or antibody (174, 175). For instance, the iCasp9 gene is often used in CAR-based cell therapy in conjunction with a small, bio-inert molecule AP1903 (Rimiducid), which acts as a chemical inducer of dimerization (CID) (200). When administered, AP1903 binds to the CID domain fused to iCasp9, leading to the formation of homodimers and subsequent activation of caspase 9. This activation triggers apoptotic cell death specifically in the CAR-engineered immune cells that express high levels of the transgene, allowing for the selective removal of inappropriately activated cells and thus providing a safety mechanism to manage potential toxicities or adverse events associated with CAR-T cell therapy (200). The efficacy of iCasp9 in eliminating CAR-engineered immune cells to counteract serious adverse event in CAR-based cell immunotherapy has been demonstrated in several preclinical studies (175, 201). Furthermore, several early phase clinical trials (NCT05239143, NCT03016377, NCT03696784 and NCT03721068) are ongoing to evaluate the safety and efficacy of this approach in patients with hematological malignancies or solid tumours, including NPC.

## 6 Efficient combinations of cellular therapies with conventional therapies

Conventional treatments such as RT and chemotherapy have long been standard for managing various malignancies. However, these approaches alone are often insufficient for eradicating large solid tumours or metastases, leading to recurrence or refractory disease. Additionally, the efficacy of immunotherapy may be restricted by an immunologically cold or immunosuppressive TME and its clinical success has primarily been limited to haematological malignancies (202, 203). Preclinical and clinical studies, however, suggested that combining conventional treatments with adoptive cell therapies can produce a synergistic anticancer effect, where RT or chemotherapy can relieve immune suppression, improve immune cell trafficking to the tumour sites, and enhance the antitumour activity of cytotoxic immune cells (204). For instance, a phase I clinical trial (NCT01462903) evaluating adoptive TIL immunotherapy following CCRT in locoregionally advanced NPC patients reported promising outcomes. Briefly, patients received RT (70 Gy) and cisplatin (100 mg/m^2^) on day 1, 22, and 43, followed by infusion of an average of 2.6 × 10^9^ TILs (range 1.3 - 6.3 × 10^9^) one week after CCRT. Of the 23 enrolled patients, 16 achieved a complete response by the end of CCRT, 19 maintained a complete response three months after adoptive cell transfer, and 18 experienced disease-free survival for over 12 months. This study demonstrated that CCRT prior to TIL infusion reduced tumour burden, decreased neutrophil and lymphocyte counts, and enhanced the expansion of EBV-antigen-specific T cells, leading to sustained antitumour activity and a robust anti-EBV immune response (84). Similarly, Chia and co-workers showed that metastatic and/or locally recurrent NPC patients who received a combination regimen of four cycles of gemcitabine and carboplatin, followed by up to six doses of EBV-specific CTLs, demonstrated a better response rate. Briefly, patients received 1000 mg/m^2^ of gemcitabine and AUC2 carboplatin on Day 1, 8, and 15 of each 28-day cycle, for a total of four cycles. This was followed by an autologous T cell infusion, with a median total CTL dose of 9.6 × 10^8^ cells (range: 6.3–10.3 × 10^8^ cells). Of the 38 patients enrolled, 35 received combination therapy. Among these patients, three achieved a complete response, 22 experienced a partial response, 10 had stable disease, and none developed progressive disease, resulting in a response rate of 71.4% compared to 42.9% during the CTL immunotherapy phase alone. Additionally, with a median follow-up of 29.9 months, the study reported a median progression-free survival of 7.6 months, surpassing the median PFS of 3.7 months only observed during the CTL immunotherapy phase alone (83).

In order to achieve this synergistic antitumour response, it is important to determine the order of administration, dosing, and volume of chemotherapy and RT when combined with cellular therapies to minimise toxicity while enhancing the efficacy of adoptive immune cells. For instance, RT can be administered prior to CAR-T cell therapy to reduce tumour burden and lessen the severity of CRS and neurotoxicity by decreasing the number of tumour cells for the CAR-T cells to target (205, 206). Poor MHC expression, low neoantigen load, and low density of infiltrating T lymphocytes are frequently associated with poor therapeutic response. Hence, in such conditions, it is necessary to upregulate the expression of neoantigens, converting the TME from cold to hot before receiving cellular therapies (207). To address this, lower doses of chemotherapy can be used to reduce immunosuppressive effects and toxicity, making it more compatible with cellular therapies. Shurin and co-workers demonstrated that low, non-cytotoxic concentrations of chemotherapeutic agents can upregulate the expression of antigen-presenting machinery components and co-stimulatory molecules on DCs, enhancing their ability to present antigens to antigen-specific T cells (208). Besides that, chemotherapy and RT have shown their ability to induce immunogenic cell death, characterised by the release of damage-associated molecular patterns (DAMPs) such as adenosine triphosphate (ATP), high mobility group box 1 protein (HMGB1), and calreticulin (CRT). These DAMPs are recognised by Toll-like receptor 4, which promotes the maturation and activation of DCs, thereby enhancing antigen presentation to CTLs and boosting the antitumour immune response (209, 210). McDonnell and co-workers also showed that systemic gemcitabine therapy can restore the capacity of suppressed or immature-like tumour-infiltrating DCs to cross-present antigens, thereby enhancing the DCs’ ability to present antigens to antigen-specific T cells and induce T cell activation (211). In the presence of high levels of immunosuppressive cells, it is recommended to deplete these cells or suppress their functions before administering cellular therapies. For instance, lymphodepleting chemotherapy with a combination of cyclophosphamide and fludarabine is usually given a few days before T cell infusion. These agents effectively eradicate Tregs and increase the production of homeostatic cytokine such as IL-15, which prolongs CAR-T cell expansion and persistence, thereby boosting their curative effects (212, 213). Chemotherapy can also sensitise tumour cells to cellular therapies. For example, chemotherapy-induced upregulation of mannose-6-phosphate receptors on the tumour cell surface enhances the penetration of T cells into the tumour site and increases the permeability of tumour cells to granzyme B in a perforin-independent manner. This increased permeability makes tumour cells, including bystander tumour cells that do not express tumour antigen, more susceptible to CTL-mediated cytotoxicity (214, 215). Similarly, Makowska and co-workers found that RT significantly increased the immunogenicity of NPC cells, leading to greater NK cell-induced killing compared to non-irradiated NPC cells. RT also upregulates the expression of PD-L1 on tumour cell surface, further enhancing the antitumour cytotoxicity of NK cells in combination with PD-L1/PD-1 blockade (78).

In the setting of NPC, it is well known that intensity-modulated radiation therapy (IMRT) alone or combined with chemotherapy has become the primary treatment for early or locally advanced patients. Hence, an effective approach to improving the homing and activation of infused immune cells, allowing their proper expansion without compromising overall immunity, would be to combine both high-dose and low-dose irradiation. Hypofractionated RT has been shown to be effective and well tolerated in patients with initial distant metastases in a phase II clinical trial (NCT03598218), compared to those who received IMRT (216). It is suggested that hypofractionated RT not only can directly kill tumour cells but also induces immunogenic cell death, releasing pro-inflammatory cytokines and DAMPs to enhance CTL-mediated cytotoxicity (217). Early preclinical studies have indeed shown that a hypofractionated regimen (8 Gy x 3) is superior to a single fraction of 20 Gy in promoting an antitumour immune response in combination with anti-CTLA-4 therapy, as evidenced by a significant increase in the number of CD4^+^ T cells and CTLs within the TME (218). Consistently, Vanpouille-Box and co-workers revealed that delivering 24 Gy in three fractions of 8 Gy promotes DC recruitment and CTL infiltration through IFN-β secretion and cGAS-STING pathway activation, enabling synergistic tumour rejection with CTLA-4 blockade therapy. However, when the single fraction dose exceeds 12 Gy, it induces the expression of DNA exonuclease Trex1, which attenuates tumour immunogenicity by degrading tumour DNA within the cytoplasm of tumour cells, leading to insufficient DC recruitment and activation of the CTL-mediated antitumour response (219). High-dose irradiation can also cause vascular damage, creating a hypoxic TME that limits CTL infiltration and leads to RT resistance (217). In contrast, low-dose RT (1 to 4 Gy) promotes immune cell infiltration without significant toxicity, reverses the suppressive function of immune cells, and inflames cold tumours, making it compatible with other anticancer treatments (220). As reported in a phase III clinical trial (NCT02456506), hyperfractionated RT significantly decreased the rate of severe adverse events and improved overall survival in patients with locally advanced recurrent NPC compared to IMRT (221). Therefore, combining a low-dose irradiation delivered in a large volume with a high dose delivered in a limited volume would improve the expansion, homing, and activation of infused T cells. Further studies are necessitated to determine optimal doses and fractionation schedules for activating the antitumour immune response while avoiding immune suppression to ensure the action of both endogenous and infused T cells.

Finally, a comprehensive evaluation and adjustment of immune cell infusion dosage are crucial for achieving optimal treatment effects. Previous studies have indicated that administering a single low dose of 2 x 10^5^ CAR-T cells/kg was effective enough in inducing a complete response with no CRS observed in patients with high tumour burden, compared to those who received a higher dose of 2 x 10^7^ CAR-T cells/kg (222). However, a high relapse rate was reported in this study, suggesting that a reduction in CAR-T cells may impair long-term efficacy. Therefore, dose fractionation or split dosing is recommended, wherein CAR-T cells are administered in multiple doses in the form of dose escalation. This approach aim to control T cell expansion and inflammatory cytokine secretion, striking a balance between long-term efficacy and safety of CAR-T cell therapy (223). Frey and co-workers compared three CAR-T cell infusion schemes in a pilot/phase I (NCT01029366) & and phase II (NCT02030847) study: high-dose single infusion (HDS, 5 x 10^8^ CAR-T cells), low-dose single or fractionated infusion (LD, 5 x 10^7^ CAR-T cells), and high-dose fractionated infusion (HDF, 5 x 10^8^ CAR-T cells). In the fractionated infusion scheme, CAR T-cells were administered over three days (Day 1, 10%; Day 2, 30%; and Day 3, 60%). Among these groups, 20 patients in the HDF cohort achieved a complete response rate of 90% with manageable CRS, compared to the HDS cohort (n = 6), where only three patients achieved complete responses, and three patients died from CRS. Although the LD cohort (n = 9) experienced manageable CRS, only 33% patients achieved complete responses. The 2-year survival rate for the HDF cohort was 73%, compared to 22% and 17% in the LD and HDS cohorts, respectively (223). Similar fractionated dosing schemes were also evaluated in another clinical trial (NCT04309981). Administration of CAR-T cells in a fractionated manner with a booster dose induced sustained responses in patients. Of the 30 patients who received the fractionated CAR-T cells dosing, 80% experienced grade 1-2 CRS, and no neurotoxic events were reported (224). Collectively, these findings suggest that fractionated dosing of CAR-T cell infusion represents a promising strategy to ensure the safety of infused T cells without compromising their efficacy. However, further studies are needed to validate this approach across different cancer types and disease burdens, as well as to optimise the timing and dosing of infusion in order to achieve long-term favourable clinical outcomes.

In summary, combining cellular therapies with existing treatment modalities for NPC involves carefully designing synergistic combinations, sequencing, and dosing strategies. Integrating conventional treatments with cellular therapies holds promise for enhancing therapeutic efficacy and overcoming resistance. However, continuous monitoring and adaptive strategies are important for optimising patient outcomes and managing potential toxicities.

## 7 Conclusions and future perspective

Despite the promising outcomes demonstrated in a vast majority of preclinical studies on cell-based therapies, there have been relatively few clinical trials conducted in NPC. This indicates that sufficient clinical evidence is still lacking to fully support the implementation of these therapies into standard clinical practice. The limitations of the existing studies include small patient sample sizes, which can lead to false-positive results and reduced statistical power. Furthermore, many of these studies are non-randomised and lack control groups, which compromises the validity and generalizability of the findings (225). Additionally, long-term toxicity data are unavailable due to the short duration of observation and post-treatment follow up. This absence of long-term data makes it challenging to assess the sustained safety and efficacy of cell-based therapies. To address these issues, future research should focus on conducting larger, randomised controlled trials with extended follow-up periods to gather comprehensive data on both the benefits and potential risks of these therapies (226). This will provide a robust evidence base to support the clinical integration of cell-based therapies for NPC.

The evolution of cancer immunotherapy has revolutionised cancer treatment, offering an alternative approach to improve the survival and quality of life for NPC patients. Immunotherapy, by redirecting effector immune cells to selectively target tumour cells, offers a significant advantage over conventional treatments like RT and chemotherapy. This approach not only enhances the host’s antitumour response but also reduces treatment-related adverse events. Likewise, the integration of cellular therapies into the treatment regimen for NPC represents a transformative shift in cancer care. Traditional treatments such as RT and chemotherapy, while effective, often come with significant toxicities and limitations. Cellular therapies offer a targeted approach to overcoming these challenges. NPC, often associated with EBV, makes this cancer a suitable candidate for cellular therapies due to its expression of potentially targetable tumour-associated viral antigens. This suitability is enhanced by the capacity to genetically engineer both stem cells and non-stem cells for specific tumour cell recognition and stable expression of a variety of antitumour agents, which holds immense clinical potential. These precision therapies can potentially lead to more effective tumour control, sparing normal tissues and reducing the systemic toxicities associated with chemotherapy and RT. By priming the immune system, cellular therapies can reduce both primary and acquired resistance and offer long-lasting protection against cancer recurrence. Engineered T cells, for example, can persist in the body, providing continuous surveillance and the capability to respond to tumour relapse. This ongoing immune surveillance can significantly improve long-term patient outcomes.

However, the clinical application of cellular therapies in solid tumours, including NPC, encounters challenges arising from tumour heterogeneity and the immunosuppressive TME, potentially compromising the therapeutic efficacy. Safety concerns, including the development of GVHD, on-target, off-tumour cytotoxicity, and CRS, present additional hurdles that ongoing and future clinical trials must effectively address. To overcome these challenges and further enhance treatment outcomes, combinatorial approaches may prove pivotal. By combining cellular therapies with existing modalities, such as conventional treatments and immunotherapy, improved efficacy in targeting cancer cells and a reduction in cancer recurrence rates can be achieved. While the exploration of cell-based therapy in NPC lags behind its application in other cancers, promising findings from published and emerging research underscore its potential to significantly improve clinical outcomes for NPC patients. Not forgetting there is a need to integrate the recent cancer discoveries, ranging from cancer immunology (227), the role of epigenetic in cancer (3, 228), novel drug delivery system (229) to increase its clinical benefits and to reduce its side effects. More comprehensive studies are therefore required to further refine the efficacy and safety of cellular therapies, paving the way for their potential integration into mainstream clinical settings for the improved management of NPC.
